# Neonatal maternal deprivation impairs localized *de novo* activity-induced protein translation at the synapse in the rat hippocampus

**DOI:** 10.1042/BSR20180118

**Published:** 2018-06-12

**Authors:** Faraz Ahmad, Mohammad Salahuddin, Khaldoon Alsamman, Hatem K. Herzallah, Sultan T. Al-Otaibi

**Affiliations:** 1 Department of Public Health, College of Public Health, Imam Abdulrahman Bin Faisal University, Dammam, Saudi Arabia; 2 Animal House Department, Institute for Research and Medical Consultations, Imam Abdulrahman Bin Faisal University, Dammam, Saudi Arabia; 3 Department of Clinical Laboratory Sciences, College of Applied Medical Sciences, Imam Abdulrahman bin Faisal University, Dammam, Saudi Arabia

**Keywords:** Akt, early-life stress, ERK1/2, maternal separation, neuropsychiatric stress, puromycin

## Abstract

Neonatal neuropsychiatric stress induces alterations in neurodevelopment that can lead to irreversible damage to neuronal physiology, and social, behavioral, and cognitive skills. In addition, this culminates to an elevated vulnerability to stress and anxiety later in life. Developmental deficits in hippocampal synaptic function and plasticity are among the primary contributors of detrimental alterations in brain function induced by early-life stress. However, the underlying molecular mechanisms are not completely understood. Localized protein translation, occurring at the synapse and triggered by neuronal activity, is critical for synapse function, maintenance, and plasticity. We used a rodent model of chronic maternal deprivation to characterize the effects of early-life neuropsychiatric stress on localized *de novo* protein translation at synaptic connections between neurons. Synaptoneurosomal preparations isolated biochemically from the hippocampi of rat pups that were subjected to maternal deprivation were deficient in depolarization-induced activity-dependent protein translation when compared with littermate controls. Conversely, basal unstimulated protein translation was not affected. Moreover, deficits in activity-driven synaptic protein translation were significantly correlated with a reduction in phosphorylated cell survival protein kinase protein B or Akt (p473 Ser and p308 Thr), but not phosphorylated extracellular signal-regulated kinase.

## Introduction

Adverse early-life experience, such as neuropsychiatric stress, induces permanent changes in the neurodevelopmental trajectory. This can lead to permanent alterations in neuronal physiology, deficits in cognitive and social skills, increased behavioral abnormalities, and vulnerability to stress and anxiety later in life [1,2]. Mother–infant interactions are a critical factor for brain development and maturation. Alterations in these interactions can result in increased susceptibility to cognitive and behavioral disorders in humans even into adulthood [2–4]. Manipulating the mother–infant relationship using maternal deprivation paradigms in rodents is a useful strategy to study the disturbances in brain function that might occur in response to adverse events during rodent neurodevelopment [1,5,6]. Adaptive responses to environmental changes, such as exposure to neuropsychiatric stress, may be detrimental to neuronal plasticity, which is driven by dynamic alterations in synaptic connectivities between the neurons and involves a complex interplay of events and molecular pathways [7]. Detrimental changes in synapse function and plasticity after exposure to early-life neuropsychiatric stress have been observed by several groups [8–10]. In addition, they are major contributors to the permanent phenotypic effects on behavior, psychology, and cognition [11–14].

The hippocampus is a limbic structure closely implicated in learning and memory function. Its neuronal circuitry is highly susceptible to activity-dependent alterations in synaptic strength. In fact, the best studied mechanisms of activity-dependent plasticity of neuronal signaling; long-term potentiation and long-term depression have been extensively studied in the hippocampus and are candidate mechanisms underpinning the higher order brain functions, such as learning and memory; the formation and modification of cognitive maps; and, importantly, the ability of organisms to react to stressful experiences [15–17]. Cognitive and neurophysiological studies suggest that the hippocampus is a primary target of endocrinological mechanisms that govern responses to neuropsychiatric stress [1,17,18]. Interestingly, there is evidence that the plasticity of neuronal circuitry in the hippocampus may increase its vulnerability to various insults, including neuropsychological stress [1,8,18–20]. Moreover, neonatal maternal deprivation can be adverse to the proper development and maturation of hippocampal neuronal circuitry [1,17]. Currently, epigenetic alterations are the most widely studied mechanisms that are thought to underlie hippocampal synaptic alterations in models of early-life stress [1,12]; however, the contribution of other nongenomic mechanisms and pathways are now beginning to be unraveled.

The presence of translational machinery and target mRNAs at synaptic terminals allows individual synapses to control the strength of their signaling independently, through neuronal activity-induced translation-dependent alterations in their synaptic protein profile [21,22]. Indeed, localized *de novo* protein translation at the synapses is critical for functional maintenance and plasticity [21,23–25]. In particular, activity-dependent synaptic protein translation plays an important role in the proper reconfiguration of neuronal circuitry [21,22,24–26]. Unsurprisingly, alterations in synaptic protein translation are acknowledged as important facet of neuronal pathologies, including neuropsychiatric disorders [21,27–30].

In the present study, we investigated the detrimental effects of maternal separation-induced neonatal stress on localized synapse-specific protein translation in the hippocampus. In addition, we analyzed levels of two principle kinases that are critical for synaptic protein translation, active phosphorylated forms of protein kinase B (PKB or Akt) and extracellular signal-regulated kinase (ERK1/2) [21,26,31–33], to determine the underlying molecular mechanisms.

## Material and methods

### Chemicals, reagents, and antibodies

Puromycin dihydrochloride (CAS No. 58-58-2) and chloramphenicol (CAS No. 56-75-7) were purchased from Millipore Merck, U.S.A. Protease and phosphatase inhibitor cocktails were from Thermo Fisher Scientific (Prod. No. 1861748, U.S.A.) and Sigma-Aldrich (Cat. No. P5726, Israel). Nylon membrane filters (100 and 10 µm) were from Merck Millipore, Ireland (Cat. Nos. NY1H02500 and NY1002500 respectively). Antibodies against PSD-95, Akt 1, pAkt 1 (Ser473), pAkt 1 (Thr308), ERK 1/2, pERK 1/2 (Thr 202/Tyr 204; Thr185/Tyr187), and α-tubulin were purchased from Thermo Fisher Scientific (Cat. Nos. MA1046, 44-654G, 700256, 710122, 700012, MA5-14898 and 322500 respectively). The antibody against puromycin was procured from Merck Millipore (Cat. No. MABE341). The horse-radish peroxidase linked secondary antibodies against rabbit (Cat. No. 31460) and mouse IgG (Cat. No. 31430) were from Thermo Fisher Scientific. Clarity™ Western ECL substrate was obtained from Bio-Rad (Cat. No. 170-5061, U.S.A.). All other chemicals purchased were of analytical grade and from either Merck Millipore or Sigma-Aldrich.

### Animals and experimental paradigm

All experiments involving animals were carried out in accordance with the institutional guidelines for animal care and use for scientific research after approval from the Institutional Review Board (IRB), Imam Abdulrahman Bin Faisal University. Female Wistar rats were housed in cages with sexually mature males (2:1; male:female ratio) under a 12/12 h light/dark cycle in rooms with a controlled temperature of 25°C and free access to food chow and drinking water. After separation from the males, each pregnant female was individually housed. On postnatal day 1 (P-1), pups were randomly assigned to maternal separation (MS) or control (Ctrl) groups. In the MS group, pups were housed separately from the dam and siblings on a heating pad at 34°C located in another room. Separation was carried out undisturbed for 3 h, from 8:00–11:00, after which the pups were returned to the home cage. In the Ctrl group, pups were housed separately from the dam for 15 min in a cage in a close proximity with the home cage to exclude the influence of “environmental handling” [34,35]. Maternal separation was performed daily from P-1 to P-21. This paradigm of daily repeated maternal deprivation is a well-established model of early-life psychological stress and has been used by several authors previously [1,35–39]. To abrogate the effects of gender-specific responses to early-life stress including maternal care deprivation, which are much less pronounced in females [40,41], only male pups were used for the study. Of note, we killed the rat pups on P-21 (which is the day when pups are weaned) immediately after the last episode of maternal separation. This has been done previously to deprive the pups of any possibility of recovery from the effects of maternal care deprivation [37]. Nevertheless, the acute contribution of the last maternal separation episode cannot be negated; however small it might be in comparison with the chronic stress to which the pups had been exposed.

### Isolation of synaptoneurosomes

A sequential filtration based protocol suitable for studying *in vitro* protein translation was employed for the biochemical isolation of synaptoneurosomes [27,42–46]. In brief, the whole hippocampi isolated from the pups were homogenized using a Potter-Elvehjem tissue grinder (Kimble, U.S.A.) in 10× volume of ice-cold translation buffer (118 mM NaCl, 4.7 mM KCl, 1.2 mM MgSO_4_, 2.5 mM CaCl_2_, 1.53 mM KH_2_PO_4_, 212.7 mM glucose, 1 mM 1,4-dithiothreitol (DTT), pH 7.4) supplemented with protease inhibitor cocktail, phosphatase inhibitor cocktail, 30 U/ml RNAse inhibitor, and 200 µg ml^−1^ chloramphenicol. The homogenate (Hgt) was sequentially passed through 100 µm (twice) and 10 µm nylon membrane filters. The filtrate obtained after passing through the two 100 µm filters was designated filtrate 1 (F1). The final filtrate obtained after passing through the 10 µm filter was centrifuged at 1500 ***g*** at 4°C for 10 min to obtain the supernatant (Sup) and the synaptoneurosomal pellet (SN). The SN pellet was resuspended in translation buffer and processed immediately for *in vitro* protein translation assay. An aliquot of the resuspended SN pellet was solubilized in SDS/PAGE sample buffer (62.5 mM Tris-HCl, pH 6.8, 2% SDS, 5% 2-mercaptoethanol, 20% glycerol, and 0.0006% Bromophenol Blue) and stored at −20°C for immunoblotting.

### Puromycin incorporation assay

Biochemically isolated subcellular preparations of synaptoneurosomes are a robust *in vitro* system for assaying local synthesis of synaptic proteins [27,42,44–46]. Moreover, synaptoneurosomes natively contain soluble factors and energy sources (synaptic mitochondria); therefore, their addition is not required in these *in vitro* samples [47]. A nonradioactive surface sensing of translation, the SunSet method [48], was utilized for the analysis of *de novo* protein synthesis in synaptoneurosomal samples. The protocol employs puromycin, a structural analog of aminoacyl tRNAs, which incorporates into nascent polypeptide chains and inhibits their elongation. When used in minimal amounts, it is possible to assess the incorporation of puromycin in polypeptides using standard immunological methods, which provides a direct measure of the protein translation rate [48,49]. This method was chosen because it has an obvious advantage over the conventional radioactivity-based S^35^-methionine incorporation assay and has been successfully used *in vitro* in both neuronal and non-neuronal systems [50–52]. In brief, synaptoneurosomes, in duplicates, were diluted with translation buffer to a concentration of 1 mg ml^−1^ protein and preincubated at 37°C for 5 min. Depolarization-induced stimulation of synaptoneurosomes was carried out by incubation with 50 mM KCl at 37°C for 15 min in the presence of 10 µg ml^−1^ puromycin. Unstimulated samples were incubated with 10 µg ml^−1^ puromycin alone. Chloramphenicol present in the medium inhibited any protein translation in the synaptic mitochondria present in the synaptoneurosomal fraction. This ensured the assay of protein translation in a synapse-specific manner [46,47]. After allowing puromycin incorporation into nascent peptide chains for 15 min, synaptoneurosomal samples were pelleted at high speed (14.3 k rpm) at 4°C, resuspended in SDS/PAGE sample buffer, and stored at −20 °C for immunoblotting.

### Immunoblotting

Synaptoneurosomal samples were separated using 4–15% gradient SDS/PAGE, electroblotted onto a polyvinylidene difluoride (PVDF) membrane, and immunostained using primary and secondary antibodies. Immunoreactive chemiluminescent signals were detected on a ChemiDoc^™^ MP Imaging System (Bio-Rad, U.S.A.), and the intensity of the bands was quantified using Image Lab software (Version 5.2, Bio-Rad, USA).

### Statistical analysis

Statistical comparisons were made by using unpaired two-tailed Student’s *t*-test. Multiple groups were compared using one-way analyses of variance (ANOVA) followed by post hoc tests with Newman–Keuls correction. The correlation between stimulated protein translation and phosphorylated Akt was calculated using Pearson’s correlation analysis. All analyses were performed using GraphPad Prism 5 software (GraphPad Software, Inc.). Results are represented as mean ± standard error of the mean (SEM) and expressed as a multiple of the respective controls. Data were considered significant if *P*<0.05.

## Results

### Biochemical preparations of synaptoneurosomes isolated from hippocampi are enriched in PSD-95

**Figure 1:**
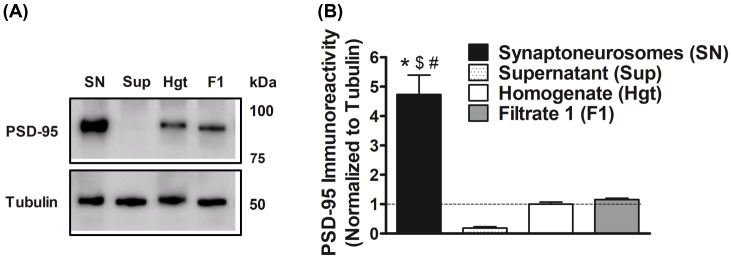
Isolation of purified synaptoneurosomal fraction from rat brain hippocampi **(A)** Immunoreactivity of postsynaptic density 95 (PSD-95), a synaptic protein marker, was analyzed to assess the purity of the synaptoneurosomes prepared by the filtration method. **(B)** PSD-95 levels in synaptoneurosomes (SN) were increased 4.7 ± 0.7 (mean ± SEM) fold when compared with homogenate (Hgt), indicating a robust enrichment of the synaptic components; Sup, supernatant obtained after the centrifugation step; F1, filtrate 1 obtained after filtration using 100 µm filters. Data are represented as mean ± SEM (*n* = 3 independent samples from three rats). *, $, and # denote statistical significance in the Sup, Hgt, and F1 groups (*P*<0.0001; *F* = 36.27; df = 3 (between columns) and 8 (within columns); ANOVA with Newman–Keuls correction).

The immunoreactivity from postsynaptic density 95 (PSD-95) expression, a synaptic marker protein [42,44], was measured in various cellular fractions to analyze the level of enrichment of the hippocampal synaptoneurosomal preparations isolated by the filtration method. PSD-95 expression in the synaptoneurosomes was 4.7 ± 1.2 (mean ± SD) fold greater than the starting homogenate material, indicating a robust enrichment of synaptic components (Figure 1).

### Depolarization-induced *de novo* localized protein translation is deficient in the synaptoneurosomes of maternally separated rat pups

**Figure 2:**
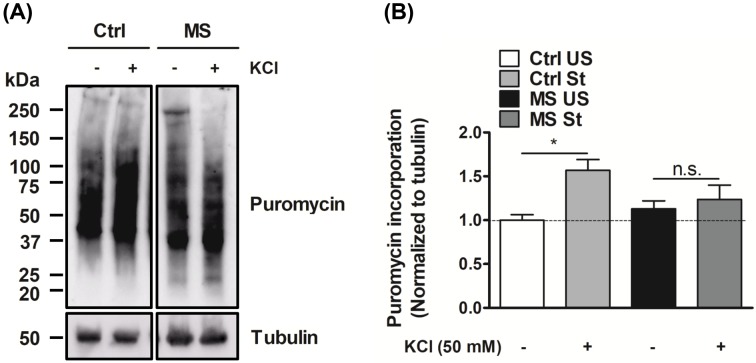
KCl-stimulated, but not basal, *de novo* protein translation is deficient in synaptoneurosomes isolated from the hippocampi of pups subjected to maternal deprivation **(A)***De novo* localized protein translation in synaptoneurosomal samples were assessed employing the non-radioactive puromycin incorporation assay follwed by immnublotting for nascent puromycin incorporated proteins. **(B)** Basal protein translation in synaptoneurosomes was unaffected in the hippocampi of MS rat pups when compared with littermate Ctrl pups. However, depolarization-induced protein translation in presence of high K^+^ was significantly diminished in the synaptoneurosomes from the hippocampi of the MS group. Data are represented as mean ± SEM (*n* = 6 rats). * denotes values significantly different from corresponding controls (*P*=0.0156; *F* = 4.403; df = 3 (between columns) and 20 (within columns); ANOVA with Newman–Keuls correction).

K^+^-mediated depolarization-induced stimulation of synaptoneurosomes was used to measure activity-dependent *de novo* protein translation [27,45,46,53]. Basal translation was evaluated in the absence of high K^+^ in the incubation medium. We found similar rates of unstimulated protein translation in the synaptoneurosomes from the Ctrl and MS groups. In addition, an increased rate of *de novo* protein synthesis after stimulation with high K^+^, was observed in Ctrl animals, as previously reported [27,45,46,53]. However, the KCl-stimulation in protein translation after depolarization was abolished in hippocampal synaptoneurosomes isolated from MS rat pups. (Figure 2).

### Phosphorylated Akt, but not phosphorylated ERK1/2, is reduced in synaptoneurosomal preparations of maternally deprived pups

**Figure 3:**
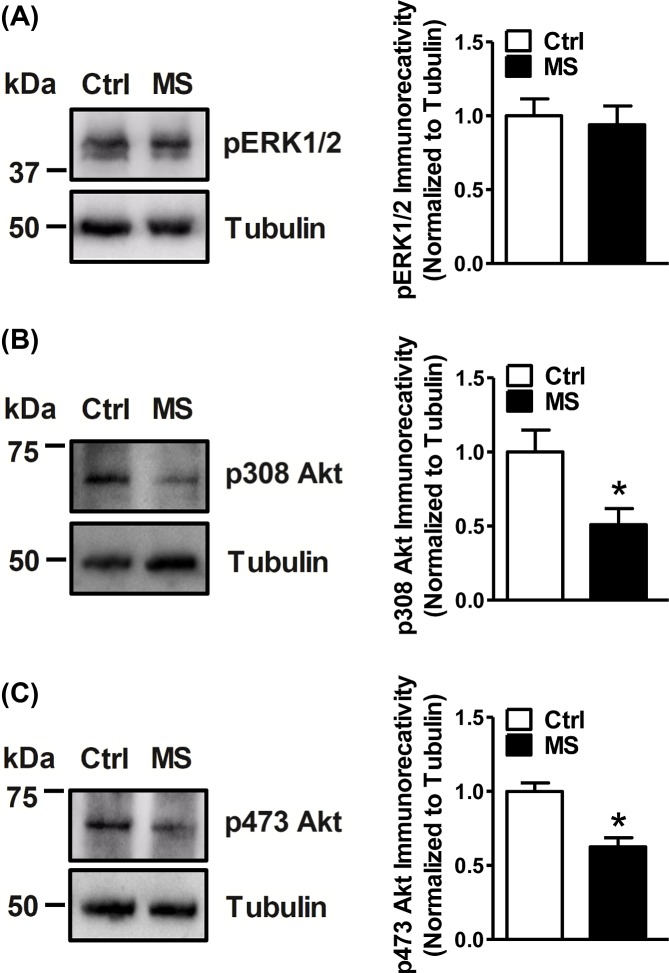
Phosphorylated Akt, but not ERK1/2, is reduced in the synaptoneurosomes of maternally deprived pups (**A**) No alteration in the immunoreactivity of phosphorylated ERK1/2 (pThr202/pTyr204; pThr185/pTyr187) was observed in synaptoneurosomes that were isolated from the hippocampi of MS rats when compared with hippocampi from littermate Ctrl rats. A significant reduction in phosphorylated Akt forms (Thr 308 (**B**)) and Ser473 (**C**)) was observed in the synaptoneurosomes of MS rats. Data are represented as mean ± SEM (*n* = 6 rats). * denotes values significantly different from corresponding controls (*P*=0.7305; *t* = 0.3542; df = 10 for (A); *P*=0.0232; *t* = 2.676; df = 10 for (B) and *P* = 0.0012; *t* = 4.483; df = 10 for (C); unpaired two-tailed Student’s *t*-test).

**Figure 4:**
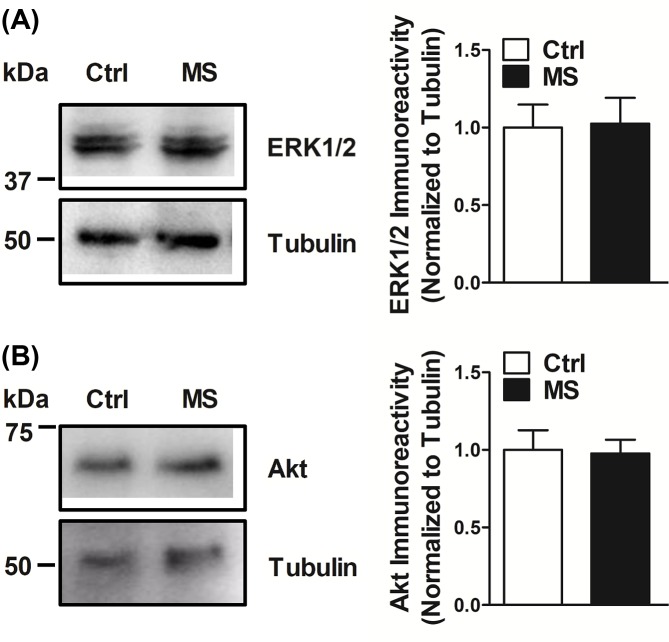
Total protein levels of Akt and ERK1/2 remain unaltered in the synaptoneurosomes of maternally deprived pups No changes were observed in the total immunoreactive levels of either ERK1/2 (**A**) or Akt (**B**) in synaptoneurosomes isolated from the hippocampi of MS rats when compared with hippocampi from littermate Ctrl rats. Data are represented as mean ± SEM (*n* = 6 rats; *P*=0.9123; *t* = 0.130; df = 10 for (A); *P*=0.8837; *t* = 0.1500; df = 10 for (B)).

Protein kinase B (PKB or Akt) and extracellular signal-regulated kinase (ERK1/2) are known modulators of local dendritic protein translation [21,26,31–33]. A complex interactive play between these kinases is thought to influence localized translational events, which in turn regulate dendritic complexity and arborization during neuroplasticity [54]. The activation of ERK is achieved by dual threonine and tyrosine residue phosphorylation (at Thr202/Tyr204 for ERK1 and Thr185/Tyr187 for ERK2). Similarly, the activation of Akt requires phosphorylation of both Thr308 and Ser473. To understand the molecular mechanism controlling the loss of activity of activity-dependent protein translation at the synapses in MS pups, we sought to assess the amount of active phosphorylated Akt and ERK1/2. While, there was no change in phospho-ERK1/2 expression in the synaptoneurosomes of MS animals when compared with littermate Ctrl pups (Figure 3A); we observed an appreciable reduction in phospho-Akt (pThr308 and pSer473 forms; Figure 3B,C) in the hippocampal synaptoneurosomes isolated from MS animals. The total Akt and ERK1/2 levels however remained unaltered (Figure 4).

### Deficits in activity-dependent protein translation correlate with the reduction in phosphorylated Akt

**Figure 5:**
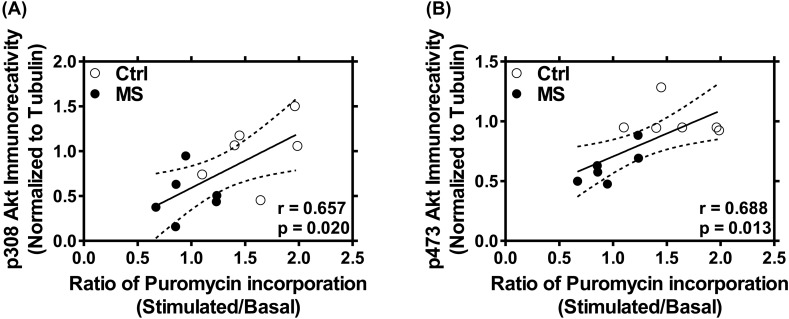
Decrease in the ratio of KCl-stimulated to basal protein translation in synaptoneurosomes correlates with reduction in levels of phosphorylated Akt Reduction in the ratio of KCl-stimulated to basal *de novo* protein translation at the synapse is significantly correlated with synaptic phosphorylated pThr 308 (**A**) and pSer 473 Akt (**B**). Data (*n* = 6 rats) were analyzed using Pearson’s correlation analysis.

Lastly, we analyzed the correlation between activity-dependent protein translation and phosphorylated Akt. There was a significant correlation between the reduction in stimulated translation in synaptoneurosomes (expressed as the ratio of KCl-induced puromycin and unstimulated basal puromycin incorporation) and the decrease in active phosphorylated Akt expression (at pThr308 and pSer473; Figure 5). These data indicate that compromises in synaptic Akt activity in pups subjected to maternal deprivation could potentially lead to translational deficits at the synapses.

## Discussion

Early stressful life increases vulnerability to social and cognitive deficits and neuropsychiatric disorders. Exposure to prolonged maternal separation can have deleterious effects on hippocampal brain functions, which manifest either immediately after stress exposure or as a long-term vulnerability to cognitive deficits in later life [6]. Hippocampal synaptic dysfunction is thought to be a primary player linking early-life stress with its adverse effects on brain function and cognition [1,8]. Alterations in the gene expression of neurotrophic factors and other critical proteins involved in synaptic signaling and plasticity, such as neurotransmitter receptors, are well-studied mechanisms of neuronal dysfunction induced by early-life stress [6]; however, it is likely that they are a single facet that is persistently altered by early-life stress. A number of other critical factors can potentially synergize with the deficient trophic support to lead to alterations in synaptic signaling and plasticity [1].

Recent studies have shown that there are several neuronal mechanisms underlying early-life stress-induced deficits in hippocampal synapse function. Neonatal maternal separation delays the GABA excitatory-to-inhibitory switch that is important for the appropriate development of neuronal signaling [55]. In addition, a reduction in α6 subunit-containing hippocampal GABA-A receptors is a key contributor to the pathophysiology of maternal separation-induced depression [56]. Alterations in the expression of cadherins, important adhesion proteins involved in remodeling synaptic connections, were observed in the hippocampi of rodents that were subjected to maternal separation [57]. Reductions in the expression of several immediate early genes, including activity-regulated cytoskeleton protein, early growth response protein 1, and glutamic acid decarboxylase, were observed in the hippocampi of maternally deprived pups. This culminates into developmental defects in spine maturation and density [58]. In another study, maternal separation-induced alterations in glycogen synthase kinase 3 beta - cAMP response element-binding protein (GSK-3β–CREB) signaling led to reductions in the expression of several plasticity-related genes [39]. Similarly, a brief daily separation model of early-life stress was found to reduce the levels of many proteins that are critical for axonal growth, myelination, and mitochondrial function [59]. Microglial activation and the consequent inflammatory response in the hippocampi of pups subjected to maternal separation have also been observed [60].

In the present study, we provide evidence for the detrimental effects of neonatal maternal separation on localized *de novo* activity-induced synaptic protein translation. Activity-dependent protein translation plays a critical role in the regulation of synaptic function and plasticity [22–26]; therefore, our discovery of early-life stress-induced dysfunction has far-reaching implications for synapse maintenance, plasticity, and memory and cognition. Moreover, synaptic plasticity and its long-term consolidation are spatiotemporally limited with the demand for both precise and dynamic changes in protein translation, trafficking, and organization within the microdomain of the synapse. Activity-driven localized protein translation affects the expression and function of many critical synaptic proteins, including neurotransmitter receptors, signaling proteins, and cytoskeletal and scaffold elements. This indicates that it is a critical contributor of the precise functioning and plasticity of synapses [26]. Furthermore, the dysregulation of plastic changes in neurotransmission through the dysfunction of the machinery used for activity-dependent synaptic translation could potentially culminate into behavioral and cognitive deficits [26,32,61].

Akt signaling is critical for activity-dependent dendritic translation [26,27,31–33]. Neuronal activity robustly stimulates local Akt phosphorylation and signaling, which allows the initiation of synaptic protein translation [28,62]. In the present study, we observed that the loss of activity-dependent translation induced by neonatal maternal separation was accompanied by diminished expression of active phosphorylated Akt (at pThr308 and PSer473 Akt) in synaptoneurosomes. Moreover, we observed a robust and significant correlation between stimulated translation and phosphorylated Akt. This indicates that Akt could be one of the primary causes of dysfunctional synaptic protein synthesis in our model of rodent maternal separation. Interestingly, basal levels of synaptic protein translation were not affected in maternally separated pups in spite of the reduction in levels of phosphorylated Akt. This indicates that basal protein translation at the synapses potentially requires only the intrinsic pAkt levels without the need for *de novo* phosphorylation of Akt. In other words, while basal pAkt levels may not be rate limiting for unstimulated protein translation, depolarization-induced protein translation requires optimal levels of pAkt including *de novo* phosphorylation at the synapses. Indeed, neuronal activity is known to robustly stimulate local Akt phosphorylation and signaling [63,64]. In contrast with the effects on phosphorylated Akt levels, our results also suggest that there is no effect of repeated maternal separation on phosphorylated ERK1/2 (pThr202/pTyr204; pThr185/pTyr187) expression. Nonetheless, our results are in agreement with those of a recent study that showed a small and transient decrease in pERK1/2 at postnatal day 7 (P-7), which was abolished at P-10 to P-21, in rat pups subjected to maternal separation [58]. Interestingly, total protein levels of both Akt and ERK1/2 at the synapses were not found to be altered upon inducing early-life stress in rat pups.

## Conclusion

We have demonstrated that early-life stress, in form of persistent exposure to neonatal maternal separation, leads to a deficiency in activity-dependent synaptic protein translation. This is mediated by a loss of active phosphorylated Akt. These data complement other studies showing a reduction in critical synaptic proteins [39,58,59] and rRNA expression [59] in models of maternal care deprivation. Our study proposes that alteration in depolarization-induced localized synaptic protein translation is a critical molecular pathway linking developmental adversities to their effects on cellular communication at the pre- and postsynapse. Furthermore, our study paves the way for further studies to determine the precise underlying mechanisms of Akt dysfunction in models of early-life stress. In addition, it opens an interesting avenue for therapeutic interventions based on Akt-stimulation to protect against the detrimental effects of early-life neuropsychiatric stress on neuronal function and cognition.

## Abbreviations

CREB: cAMP response element-binding protein
ERK1/2: extracellular signal-regulated kinase
GSK-3b: glycogen synthase kinase 3 beta
PKB: protein kinase B
PSD-95: postsynaptic density 95
